# IQGAP1 promotes anoikis resistance and metastasis through Rac1-dependent ROS accumulation and activation of Src/FAK signalling in hepatocellular carcinoma

**DOI:** 10.1038/s41416-020-0970-z

**Published:** 2020-07-07

**Authors:** Chun-fen Mo, Jun Li, Shu-xia Yang, Hui-jie Guo, Yang Liu, Xing-yan Luo, Yan-tang Wang, Min-hui Li, Jing-yi Li, Qiang Zou

**Affiliations:** 1 Department of Immunology, School of Basic Medical Sciences, Chengdu Medical College, Chengdu, China; 2 Department of Gastroenterology, The first affiliated hospital of Chengdu medical college, Chengdu, China; 3 Department of Urology, The Second Affiliated Hospital of Chengdu Medical College, China National Nuclear Corporation 416 Hospital, Chengdu, China; 4 School of Biological Sciences and Technology, Chengdu Medical College, Chengdu, China

**Keywords:** Metastasis, Cell signalling

## Abstract

**Background:**

Hepatitis B virus (HBV) has a crucial role in the progression of hepatocellular carcinoma (HCC). Tumour cells must develop anoikis resistance in order to survive before metastasis. This study aimed to investigate the mechanism of IQGAP1 in HBV-mediated anoikis evasion and metastasis in HCC cells.

**Methods:**

IQGAP1 expression was detected by immunohistochemistry, real-time PCR and immunoblot analysis. Lentiviral-mediated stable upregulation or knockdown of IGAQP1, immunoprecipitation, etc. were used in function and mechanism study.

**Results:**

IQGAP1 was markedly upregulated in HBV-positive compared with HBV-negative HCC cells and tissues. IQGAP1 was positively correlated to poor prognosis of HBV-associated HCC patients. IQGAP1 overexpression significantly enhanced the anchorage-independent growth and metastasis, whereas IQGAP1-deficient HCC cells are more sensitive to anoikis. Mechanistically, we found that HBV-induced ROS enhanced the association of IQGAP1 and Rac1 that activated Rac1, leading to phosphorylation of Src/FAK pathway. Antioxidants efficiently inhibited IQGAP1-mediated anoikis resistance and metastasis.

**Conclusions:**

Our study indicated an important mechanism by which upregulated IQGAP1 by HBV promoted anoikis resistance, migration and invasion of HCC cells through Rac1-dependent ROS accumulation and activation of Src/FAK signalling, suggesting IQGAP1 as a prognostic indicator and a novel therapeutic target in HCC patients with HBV infection.

## Background

Hepatocellular carcinoma (HCC) is one of the mostly common malignancies and the second leading cause of cancer-related deaths worldwide.^1^ Chronic hepatitis B virus (HBV) infection, which can cause both acute and chronic liver disease, is one of the major risk factors for HCC development. It is estimated that there are 350 million people chronically infected with HBV and nearly one million people die every year due to complications of HBV infection.^2^ Curative therapies including local ablative therapy, surgical resection and liver transplantation improved survival in HCC patients; however, a large proportion of patients with advanced stages of HCC will develop metastasis and the natural survival time of these patients is only ~3 months.^3,4^ Therefore, understanding the mechanisms of HBV-associated HCC pathogenesis will develop new strategies to improved prediction, prevention and treatment of HCC.

The extracellular matrix (ECM), a part of the extracellular environment, provides adhesive support and elicits signal transduction that regulates cell proliferation, migration, differentiation and survival.^5^ Anoikis is a form of anchorage-dependent programmed cell death caused by the loss of cell-matrix adhesion.^6^ Anoikis resistance is a key feature of metastatic cancers that confers anchorage-independent growth of cancer cells to local dissemination and distant colonisation.^7^ A recent study has confirmed that hepatitis B virus X (HBx) protein endues resistance of HCC cells to anoikis through upregulation and activation of p21-activated kinase 1.^8^ Reactive oxygen species (ROS), which are physiologically generated continuously by cellular metabolism, play an important role in the survival of ECM-detached cancer cells.^9,10^ Moreover, HBV infection is accompanied by the induction of oxidative stress in host cells, and ROS accumulation is necessary for HBV replication.^11,12^ HBx protein activates STAT3 and NF-κB signalling and induces mitochondrial translocation of Raf-1 that is mediated by ROS.^13^ In addition, antioxidant molecules such as glutathione peroxidase, superoxide dismutase and catalase dramatically decreased HBx levels through inhibition of intramolecular and intermolecular disulfide bonds formation of HBx protein.^14^ However, the molecular mechanism by which ROS contribute to HBV-mediated anoikis resistance and metastasis requires further clarification.

IQ domain GTPase-activating protein 1 (IQGAP1) is a scaffolding protein that regulates extracellular signals and cellular motility through interacting with cytoskeletal, cell adhesion and signal transduction proteins including calmodulin, β-catenin and Cdc42.^15–17^ Although IQGAP1 contains a domain that is similar to Ras-GAPs, IQGAP1 does not act as a traditional GAP. For example, IQGAP1 can stabilise Cdc42 in its GTP-bound form, thereby increasing active Cdc42.^18,19^ High levels of IQGAP1 are observed in various tumours. Notably, upregulated IQGAP1 levels are detected in tumour budding foci at the invasive front of colorectal and ovarian tumours compared to central tumour regions and normal tissues,^20,21^ suggesting a critical role of IQGAP1 in tumour invasion and metastasis. However, it remains unknown whether IQGAP1 is involved in HBV-mediated HCC progression.

Here, we showed that IQGAP1 was upregulated in HBV-positive compared with HBV-negative HCC cells and tissues. High level of IQGAP1 was closely related to poor prognosis of HBV-associated HCC patients. Enforced IQGAP1 expression significantly enhanced the anchorage-independent growth, migration and invasion of HCC cells, whereas IQGAP1-deficient HCC cells are more sensitive and vulnerable to anoikis. Mechanistically, we demonstrated that HBV augmented the association of IQGAP1 and Rac1, leading to increased intracellular levels of ROS that subsequently accelerated the phosphorylation of Src kinase, and ultimately activated FAK signalling. Collectively, our findings revealed that IQGAP1 promoted HBV-mediated anoikis resistance and metastasis through Rac1-dependent ROS accumulation and activation of Src/FAK pathway, implicating IQGAP1 as both a potential therapeutic target and a predictor of survival in HBV-associated HCC patients.

## Methods

### Cell culture and reagents

HepG2, HepG2.2.15 (which stably transfect HBV genome^22^) and Huh7 cells were maintained in Dulbecco’s modified Eagle’s medium (DMEM) supplemented with 10% foetal bovine serum and 1% antimycotic at 37 °C in an atmosphere of 5% CO_2_. HepAD38 cells (which replicate HBV under tetracycline-off control^23^) were cultured in DMEM medium supplemented with 400 μg/ml G418 and 2 μg/ml tetracycline as described previously.^24^ Withdrawal of tetracycline from the medium induces HBV replication in HepAD38 cells.

The pcDNA3-EGFP-Rac1 (WT) (Addgene plasmid #13719), pcDNA3-EGFP-Rac1 (Q61L) (Constitutively active mutants, Addgene plasmid #13720), and pcDNA3-EGFP-Rac1 (T17N) (Dominant-negative mutants, Addgene plasmid #13721) were gifts from Klaus Hahn. The following reagents were used: FAK Inhibitor 14 (SML0837, Sigma), H_2_O_2_ (323381, Sigma), NAC (A9165, Sigma), Protease inhibitor cocktail (P8340, Sigma), Dithiothreitol (D9779, Sigma), PP2 (P0042, Sigma), Trypan Blue (T6146, Sigma), siRNA targeting Src (s13414, Thermo), FAK (s11485, Thermo) and Negative Control (4390846, Thermo).

### Establishment of stable IQGAP1-overexpressing and -knockdown HCC cells

The lentiviral vector (GV358) for overexpression of IQGAP1 was constructed by inserting IQGAP1 cDNA sequences (NM_003870.3). Two specific shRNAs (5’-CAA CGA CAT TGC CAG GGA TAT-3’ and 5’-ATC AGG ACC TGC TGC AGC TAC-3’), each targeting a distinct sequence of human IQGAP1, were used to knockdown IQGAP1 and cloned into a lentivirus shuttle vector (GV248). Both GV358 and GV248 vectors expressed EGFP. The production of the recombinant lentiviral was conducted by Genechem Company (Shanghai, China). The cells were transduced with lentiviral and selected with puromycin for 14 days.

### Quantitative real-time polymerase chain reaction (qRT-PCR)

Total RNA was extracted using RNAiso Plus (TAKARA) according to the manufacturer’s instructions as described previously.^25^ The qRT-PCR was conducted on the CFX96 real-time PCR system (Bio-Rad, USA). All reactions were performed by using SYBR^®^ Premix Ex Taq™ II (TAKARA). The following primers for qRT-PCR were selected: IQGAP1,5’-CAG AGA AGA TTG GCA GCA GTA GC-3’ and 5’- GAG CTC TGG GTG GGT GAG ATT A-3’; GAPDH, 5’-TCC ACC ACC CTG TTG CTG TA-3’ and 5’-ACC ACA GTC CAT GCC ATC AC-3’.

### Immunoprecipitation and immunoblot analysis

Immunoprecipitations were performed essentially as described previously.^26^ Briefly, the cells were washed with ice-cold PBS and then homogenised in RIPA lysis buffer containing protease inhibitors on ice for 30 min. Equal amounts of protein lysates were incubated with indicated antibodies at 4 °C overnight. Immune complexes were collected by protein A Sepharose beads (GE Healthcare), separated by SDS-PAGE, and transferred on to PVDF membranes (Millipore). The membranes were blocked for 1 h at room temperature with 5% non-fat milk in TBS buffer and incubated at 4 °C overnight with indicated primary antibodies. The following antibodies were used: Src (#2123, Cell Signaling Technology), cleaved PARP (Cell signalling Technology, #5625), p-Src (#6943, Cell Signaling Technology), p-FAK (#8556, Cell Signaling Technology), Rac1 (#8631, Cell signaling Technology), cleaved caspase-3 (Cell Signaling Technology, #9664), FAK (#13009, Cell Signaling Technology), IQGAP1 (ab86064, Abcam), FLAG (F7425, Sigma) and β-actin (sc-47778, Santa Cruz Biotechnology). The immunoblot signals were detected with the Immobilon^TM^ Western Chemiluminescent HRP Substrate (Millipore). The relative densitometric analyses of western blotting images were performed by Image J software as described in the website of the University of Queensland (https://di.uq.edu.au/community-and-alumni/sparq-ed/sparq-ed-services/using-imagej-quantify-blots).

### Rac1 activity assay

Rac1 activity assay was examined using the Active Rac1 Detection kit as described previously.^27^

### Anoikis assays in vitro

HCC cells were resuspended in normal DMEM medium and placed on poly-HEMA-coated plates to prevent cell adhesion. The addition of growth factor-reduced Matrigel (BD Biosciences) to medium restores the integrin signalling in suspension as described previously.^28^ The cell death was assessed by trypan blue exclusion assay.

### In vivo anoikis assay

Female BALB/c nu/nu mice (6 week old) were purchased from the Chengdu Dashuo Biotechnology Corporation and raised in specific pathogen-free conditions. All animal experiments were carried out at the Animal Center of Chengdu Medical College. Animal experimental procedures were approved by the Institutional Animal Care and Use Committee of Chengdu Medical College. For the experimental mouse anoikis metastasis model, the indicated HCC cells (2 × 10^6^ cells/100 μl) were injected into nude mice (five mice each group) through the tail vein. The mice were monitored daily and euthanised by CO_2_ asphyxiation about 8 weeks after injection to examine the lung metastasis of tumour cells. The tumour metastases were confirmed by H&E staining and quantified based on visual examination and manual counting of formalin-fixed lungs. For mouse peritoneal cavity model, indicated HCC cells (5 × 10^6^ cells/100 μl) were intraperitoneally injected into nude mice as described previously.^29^ When tumour cells developed detectable ascites, mice were randomly divided into four groups (five mice each group). PP2 (5 mg/kg) or Y15 (30 mg/kg) was dosed by intraperitoneal injection daily, and the control group received an equal volume of vehicle. After treatment for 72 h, the mice were euthanised by CO_2_ asphyxiation, and ascites fluid was collected and centrifuged. GFP-positive cells were sorted by flow cytometer and analysed by trypan blue exclusion assay.

### Statistical analysis

All quantitative data were presented as the mean ± SD. Student’s *t*-test and one-way ANOVA test were used to compare quantitative variables where appropriate. Pearson χ^2^ test was used to compare qualitative variables. Kaplan–Meier analysis with log-rank test was used to calculate the patients’ survival rates. Statistical analyses were performed using SPSS software version 22.0. *P*-value < 0.05 was considered to be statistically significant.

## Results

### Upregulation of IQGAP1 by HBV infection correlates with malignant progression and poor prognosis of HBV-associated HCC patients

**Fig. 1:**
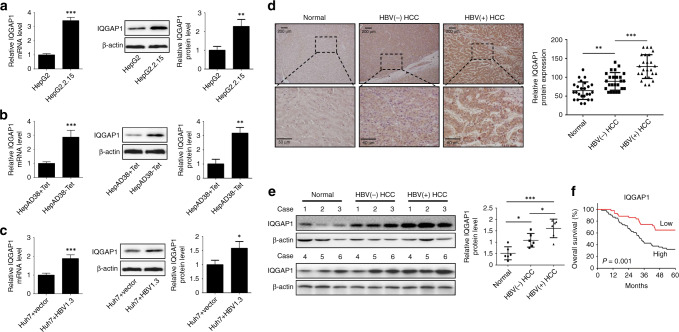
IQGAP1 is upregulated in HBV-associated HCC cells and tissues. **a**, **b** The qRT-PCR and immunoblot analysis of IQGAP1 levels in HBV-positive HCC cells (HepG2.2.15 and HepAD38-Tet) and HBV-negative cells (HepG2 and HepAD38+Tet). **c** IQGAP1 levels in Huh7 cells transfected with HBV1.3 or vector plasmid were determined by qPCR and immunoblot analysis. **d** Representative images of IQGAP1 expression in clinical normal and HCC tissues obtained by immunohistochemical analysis. **e** Immunoblot analysis of IQGAP1 protein levels in adjacent normal, HBV-negative and HBV-positive tissues of HCC patients. **f** The overall survival curves of HBV-associated HCC patients with low- or high-IQGAP1 expression levels. Data were mean ± standard deviation (SD) from at least three independent experiments. **P < 0.01 and ***P < 0.001.

To clarify the role of IQGAP1 in HBV-induced HCC, we first detected IQGAP1 levels in different HCC cell lines. As shown in Fig. 1a, both the mRNA and protein expressions of IQGAP1 were markedly increased in HBV-producing cell line HepG2.2.15 than HepG2 cell. Likewise, higher IQGAP1 expressions were observed in non-tetracycline-treated HepAD38 cells compared to tetracycline-treated HepAD38 cells (Fig. 1b). Moreover, Huh7 cells transfected with the 1.3-fold HBV replicative genome (HBV1.3) plasmids showed elevated expressions of IQGAP1 (Fig. 1c). Next, we determined the expression of IQGAP1 protein in HCC patients. Immunohistochemical analysis demonstrated that IQGAP1 protein was predominantly localised in the cytoplasm in adjacent normal tissues, and with partial nuclear staining in HCC tissues (Fig. 1d). IQGAP1 protein was increased in HCC tissues compared to adjacent normal tissues (Fig. 1d). Notably, higher levels of IQGAP1 were observed in HBV-positive than HBV-negative HCC tissues (Fig. 1d). Similar results were verified by immunoblotting analysis (Fig. 1e). Next, we investigated IQGAP1 level in HCC tissues using the TCGA database. TCGA liver cancer dataset showed that there was no significant difference between IQGAP1 mRNA levels in normal and HCC tissues, while IQGAP1 mRNA was upregulated in HBV-positive compared to HBV-negative HCC tissues (Supplementary Fig. S1a). These results implied that IQGAP1 protein stability may be dysregulated during HCC progression. To test this probability, immortal hepatocyte cell line LO2, HCC cells HepG2 and HepG2.2.15 were treated with a eukaryote protein synthesis inhibitor Cycloheximide, respectively. We found decreased degradation rate of IQGAP1 protein in HepG2 and HepG2.2.15 cells compared to LO2 cells (Supplementary Fig. S1b). Moreover, IQGAP1 protein in HepG2.2.15 cells was more stable than that in HepG2 cells, suggesting that HBV infection increased IQGAP1 protein stability (Supplementary Fig. S1b). Thus, we speculated that HBV could promote IQGAP1 upregulation through enhancing the transcription and protein stability of IQGAP1. Based on the clinicopathological information, we found that a higher level of IQGAP1 expression was positively correlated with HBsAg and AFP level, tumour size and number and BCLC stage (Table 1). HBV-associated HCC patients were grouped according to the mean value of IQGAP1 expression, and we found that HCC patients with high-IQGAP1 expression had shorter overall survival when compared with the low-IQGAP1-expressing group (Fig. 1f). Therefore, these data indicate that increased IQGAP1 expression caused by HBV infection is associated with the malignant progression and poor prognosis of HCC patients.

**Table 1:** Correlation between the clinicopathological characteristics and IQGAP1 in HCCl.

Characteristic	High-IQGAP1 expression (*n* = 58)	Low-IQGAP1 expression (*n* = 56)	*p*-value
Age			0.638
≤60	38 (49.4%)	39 (50.6%)	
>60	20 (54.1%)	17 (45.9%)	
Gender			0.342
Male	44 (53.7%)	38 (46.3%)	
Female	14 (43.8%)	18 (56.2%)	
HBsAg			0.002
Negative	6 (24.0%)	19 (76.0%)	
Positive	52 (58.4%)	37 (41.6%)	
AFP (ng/ml)			0.025
≤200	19 (38.8%)	30 (61.2%)	
>200	39 (60.0%)	26 (40.0%)	
Differentiation			0.146
Well	6 (50.0%)	6 (50.0%)	
Moderate	30 (44.1%)	38 (55.9%)	
Poor	22 (64.7%)	12 (35.3%)	
Tumour size			0.001
≤5 cm	12 (29.3%)	29 (70.7%)	
>5 cm	46 (63.0%)	27 (37.0%)	
Tumour number			0.034
Solitary	28 (42.4%)	38 (57.6%)	
Multiple	30 (62.5%)	18 (37.5%)	
BCLC stage			0.005
0–A	20 (37.0%)	34 (63.0%)	
B–C	38 (63.3%)	22 (36.7%)	
Cirrhosis			0.552
No	8 (44.4%)	10 (55.6%)	
Yes	50 (52.1%)	46 (47.9%)	

### IQGAP1 enhances anoikis resistance and metastasis

**Fig. 2:**
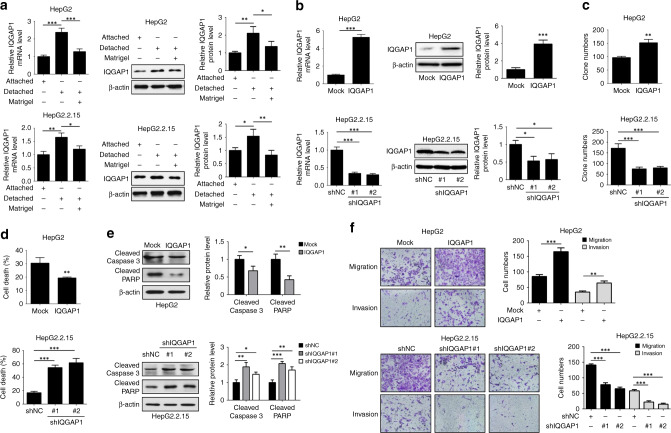
IQGAP1 promotes anoikis resistance, migration and invasion of HCC cells. **a** The qRT-PCR and western blotting analysis of IQGAP1 levels in attached or detached HCC cells treated with or without Matrigel. **b** The efficiency of stable upregulation of IQGAP1 in HepG2 cells and stable downregulation of IQGAP1 in HepG2.2.15 cells was validated by qRT-PCR and western blotting analysis. **c** The anchorage-independent growths of indicated HCC cells were determined by soft agar colony formation assays. **d** The cell death of indicated HCC cells under suspension condition was determined by trypan blue exclusion assay. **e** Indicated HCC cells were placed on poly-HEMA-coated plates to prevent cell adhesion, and then followed by western blotting analysis with indicated antibodies. **f** Indicated HCC cells were subjected to transwell migration and invasion assays. The results were expressed as mean ± SD from at least three independent experiments. *P < 0.05, **P < 0.01 and ***P < 0.001.

To assess whether IQGAP1 contributes to HBV-induced anoikis resistance, we examined the expression of IQGAP1 in HepG2 and HepG2.2.15 cells following detachment. IQGAP1 mRNA and protein levels were increased in HepG2 and HepG2.2.15 cells after detachment (Fig. 2a). However, the addition of Matrigel to detached HCC cells mitigated the elevated expressions of IQGAP1, suggesting a potential correlation between IQGAP1 and anoikis (Fig. 2a). Next, we ectopically expressed IQGAP1 in non-HBV-producing HepG2 cells and knocked down IQGAP1 in HBV-producing HepG2.2.15 cells, respectively (Fig. 2b). Enforced IQGAP1 expression markedly promoted anchorage-independent growth of HepG2 cells, while repression of IQGAP1 reduced the resistance to anoikis in HepG2.2.15 cells (Fig. 2c). Moreover, IQGAP1 silence substantially inhibited the increased anchorage-independent growth of Huh7 cells transfected with HBV1.3 plasmids (Supplementary Fig. S2a). The similar phenomenon was confirmed by trypan blue exclusion test (Fig. 2d and Supplementary Fig. S2b). The upregulation of IQGAP1 effectively reduced the expressions of cleaved caspase-3 and cleaved PARP in HepG2 cells during ECM detachment (Fig. 2e). The converse results were observed in IQGAP1-defcient HepG2.2.15 cells (Fig. 2e). In addition, HBV replication induced the reduced activation of caspase-3 and PARP that was inversed by IQGAP1 knockdown after detachment (Supplementary Fig. S2c). As shown in Fig. 2f, overexpression of IQGAP1 significantly enhanced the migratory and invasive capacities of HepG2 cell, whereas IQGAP1 suppression in HepG2.2.15 cells caused a significant decline in cell migration and invasion. These data imply that IQGAP1 facilitates anoikis resistance and metastasis in HCC cells.

### IQGAP1-induced anoikis resistance and metastasis is dependent on ROS accumulation

**Fig. 3:**
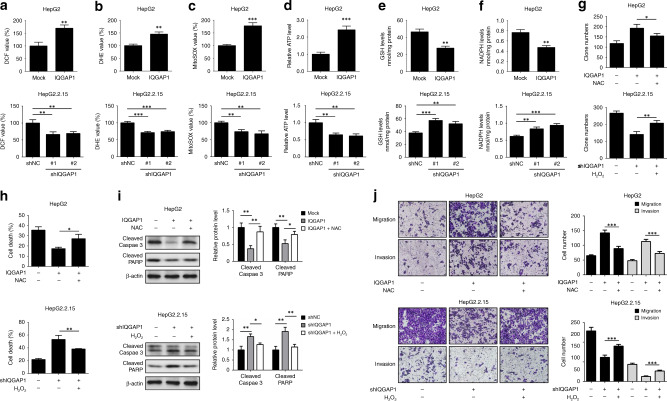
ROS are required for IQGAP1-mediated anoikis resistance and metastasis in HCC cells. **a**, **b** The effect of IQGAP1 on ROS production in HCC cells was determined by DCF and DHE assay. **c** The mitochondrial ROS levels in indicated HCC cells were examined by MitoSOX Red staining assay. **d**–**f** The indicated HCC cells were maintained in suspension condition, and then intracellular ATP (**d**), reduced GSH (**e**) and NADPH (**f**) levels were measured. **g** Indicated HepG2 and HepG2.2.15 cells were exposed to 20 mM NAC or 10 μM H_2_O_2_, respectively, and then subjected to soft agar colony formation assays. **h** Indicated HCC cells were maintained in suspension condition, treated as in **g**, and then followed by trypan blue assay. **i** Indicated HCC cells were maintained, treated as in **g**, and analysed by western blotting with indicated antibodies. **j** Indicated HCC cells were treated as in **g**, and then subjected to transwell migration and invasion assays. The results were expressed as mean ± SD from at least three independent experiments. *P < 0.05, **P < 0.01 and ***P < 0.001.

As oxidative stress has a critical role in HBV-induced HCC progression and anoikis resistance, we next determined whether IQGAP1 could affect ROS production in HCC cells. Compared with control, both H_2_O_2_ and O_2_^−^ levels were increased in IQGAP1-overexpressing HCC cells (Fig. 3a, b). However, IQGAP1-deficient cells showed less ROS levels (Fig. 3a, b). Similar results were observed in mitochondrial ROS assay (Fig. 3c). Moreover, upregulated IQGAP1 caused elevated ATP generation, while IQGAP1 suppression diminished ATP production (Fig. 3d). The levels of two major redox buffers GSH and NADPH were decreased in IQGAP1-overexpressing HepG2 cells under detachment conditions (Fig. 3e, f). Conversely, IQGAP1-knockdown cells exhibited higher GSH and NADPH amounts (Fig. 3e, f). These results imply that IQGAP1 increased ROS accumulation and ATP production, but reduced antioxidative capacity in detached HCC cells.

To further elucidate the involvement of ROS in IQGAP1-mediated anoikis resistance and metastasis, HepG2 and HepG2.2.15 cells were treated with oxidant agent H_2_O_2_ or antioxidant agent NAC in a dose-dependent manner, respectively. Indeed, the anchorage-independent growth of HepG2 cells was obviously increased following lower-dose H_2_O_2_ treatment (10 and 20 μM), however, higher doses of H_2_O_2_ (more than 50 μM) resulted in cytotoxic efect (Supplementary Fig. S3a). NAC treatment induced markedly increased apoptosis of HepG2.2.15 cells after detachment (Supplementary Fig. S3b), suggesting that modest levels of ROS act as indispensable roles for maintaining cell survival in response to detachment. Next, we determine whether ROS were required for IQGAP1 function. As shown in Fig. 3g, the protective effect of IQGAP1 overexpression against anoikis was reversed by 20 mM NAC treatment in HepG2 cells, while 10 μM H_2_O_2_ treatment partially restored the anchorage-independent growth in IQGAP1-deficient HepG2.2.15 cells. NAC administration resulted in decreased survival and increased cleaved caspase-3 and cleaved PARP protein levels of HepG2 cells in the presence of IQGAP1, whereas exogenous addition of H_2_O_2_ significantly attenuated the elevated cell death rate and activation of caspase-3 and PARP in IQGAP1-silenced HepG2.2.15 cells upon detachment (Fig. 3h, i). In addition, transwell assay showed that NAC compromised the capacity of IQGAP1 to promote migration and invasion (Fig. 3j). Conversely, H_2_O_2_ effectively rescued the effect of IQGAP1 knockdown on the motility of HepG2.2.15 cells (Fig. 3j). Our results demonstrate that ROS accumulation is required for the development of IQGAP1-mediated anoikis resistance and metastasis in HCC cells.

### The activation of Rac1 is essential for IQGAP1-mediated anoikis resistance and metastasis

Because IQGAP1 has been shown to bind of Rac1,^19^ we examined whether HBV altered the interaction of IQGAP1 with Rac1. The amounts of Rac1 co-immunoprecipitated with IQGAP1 were markedly increased in HepG2.2.15 cells compared to HepG2 cells (Supplementary Fig. S4a). Consistently, transient transfection of Huh7 cells with HBV1.3-fold genome also promoted the association of IQGAP1 with Rac1 (Supplementary Fig. S4b). Moreover, we found that the elevated levels of GTP-bound Rac1 were observed in IQGAP1-overexpressing HepG2 cells, while IQGAP1 suppression abrogated the capability of HBV to activate Rac1 in HepG2.2.15 cells (Supplementary Fig. S4c). Next, we investigated the effect of ROS on IQGAP1-mediated Rac1 activation. Immunoprecipitation assay showed that the interaction between IQGAP1 and Rac1 was enhanced in response to H_2_O_2_ stimulus, whereas NAC treatment repressed IQGAP1 binding to Rac1 both in HepG2 and HepG2.2.15 cells (Supplementary Fig. S4d). Besides, NAC significantly compromised the capacity of IQGAP1 to activate Rac1 (Supplementary Fig. S4e). In contrast, reduced Rac1 activity caused by IQAGP1-knockdown was partially alleviated by H_2_O_2_ in HepG2.2.15 cells (Supplementary Fig. S4e). Our results suggest that IQGAP1 contributes to the activation of Rac1, and this procedure is regulated by ROS.

**Fig. 4:**
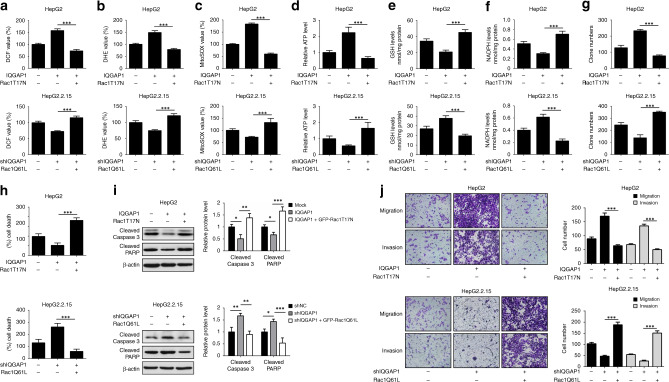
Rac1 acivation is essential for IQGAP1-mediated ROS production, anoikis resistance and metastasis in HCC cells. **a**, **b** Indicated HCC cells were transfected with EGFP-Rac1T17N (dominant-negative Rac1 mutant) or EGFP-Rac1Q61L (dominant-active Rac1 mutant) plasmid, respectively. The production of intracellular ROS was determined by DCF and DHE assay. **c** The mitochondrial ROS levels in indicated HCC cells were examined by MitoSOX Red staining assay. **d**–**f** Indicated HCC cells were transfected, maintained in suspension condition and then intracellular ATP (**d**), reduced GSH (**e**) and NADPH (**f**) levels were measured. **g** Indicated HCC cells were transfected with EGFP-Rac1T17N or EGFP-Rac1Q61L plasmid, respectively. Soft agar colony formation assays were performed. **h** Indicated HCC cells were transfected as in **g**, maintained in suspension condition, and then followed by trypan blue assay. **i** Indicated HCC cells were transfected, maintained as in **g**, and analysed by western blotting with indicated antibodies. **j** Indicated HCC cells were transfected as in **g**, and then followed by transwell assays. The results were expressed as mean ± SD from at least three independent experiments. ***P < 0.001.

To further evaluate the effect of Rac1 in IQGAP1-mediated HCC progression, IQGAP1-overexpressing HepG2 cells were transfected with Rac1T17N (dominant-negative mutant) plasmid and IQGAP1-deficient HepG2.2.15 cells were transfected with Rac1Q61L (constitutively active mutant) plasmid. The efficiency of these plasmids was validated by immunoblot analysis (Supplementary Fig. S5). Our results showed that overexpression of Rac1T17N attenuated the increased ROS levels caused by IQGAP1 in HepG2 cells (Fig. 4a–c). Conversely, transfection of Rac1Q61L in IQGAP1-knockdown HepG2.2.15 cells has the opposite effect (Fig. 4a–c). Rac1T17N dramatically reversed the elevated ATP generation triggered by upregulated IQGAP1 in HepG2 cells. In contrast, Rac1Q61L overcame decreased intracellular ATP levels caused by IQGAP1 silencing (Fig. 4d). Moreover, IQGAP1-induced reduced contents of GSH and NADPH were significantly prevented by Rac1T17N mutant in HepG2 cells, while Rac1Q61L mutant resulted in obviously suppression of GSH and NADPH levels in IQGAP1-defcient HepG2.2.15 cells under detachment conditions (Fig. 4e, f). In addition, Rac1T17N mutant eliminated IQGAP1-induced the enhanced anchorage-independent growth of HepG2 cells, while Rac1Q61L mutant rescued the anoikis resistance of IQGAP1-deficient HepG2.2.15 cells (Fig. 4g, h). Similar results were observed in immunoblot analysis and transwell assay (Fig. 4i, j). These observations indicate that Rac1 is a pivotal downstream effector of IQGAP1 to regulate anoikis evasion and metastasis in HCC cells.

### IQGAP1 augments anoikis resistance and metastasis through activating Src/FAK pathway

**Fig. 5:**
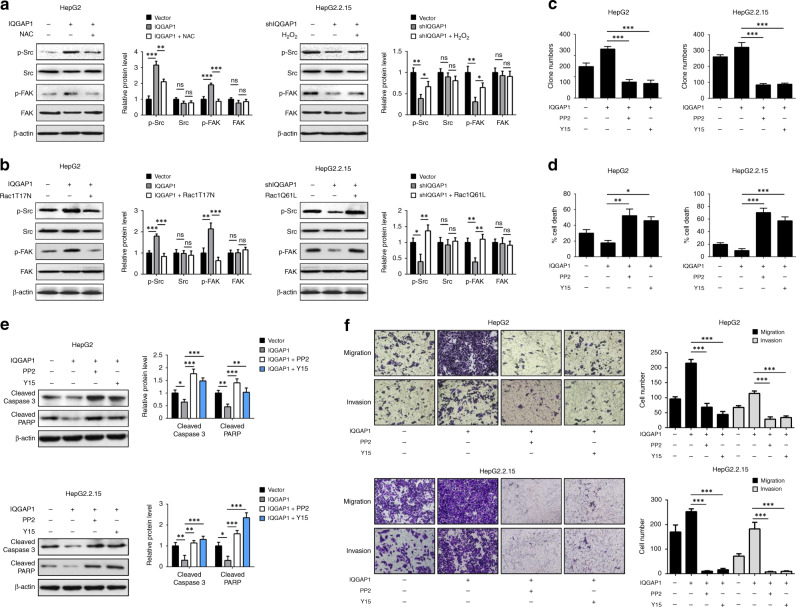
IQGAP1 activates Src/FAK signalling pathway. **a** Indicated HCC cells maintained in suspension condition were exposed to 20 mM NAC or 10 μM H_2_O_2_, respectively. After incubation for 48 h, immunoblot analysis was performed with indicated antibodies. **b** Indicated HCC cells were transfected with EGFP-Rac1T17N or EGFP-Rac1Q61L plasmid, respectively, and then analysed by western blotting with indicated antibodies. **c** HepG2 and HepG2.2.15 stably expressing IQGAP1 were treated with Y15 or PP2, and then followed by soft agar colony formation assays. **d** Indicated HCC cells were maintained in suspension condition, treated as in **c** and then followed by trypan blue assay. **e** Indicated HCC cells were maintained, treated as in **d** and analysed by western blotting with indicated antibodies. **f** Indicated cells treated with Y15 or PP2 were subjected to transwell migration and invasion assays. The results were expressed as mean ± SD from at least three independent experiments. *P < 0.05, **P < 0.01 and ***P < 0.001.

Accumulating evidence demonstrated that the activation of Src/FAK signalling was involved in the aggressive progression of HCC.^30–32^ To gain insight into the molecular mechanisms by which IQGAP1 promotes anoikis resistance and metastasis, we determined whether IQGAP1 could activate Src/FAK signalling. Ectopic expression of IQGAP1 increased Src Tyr^416^ and FAK Tyr^397^ in HepG2 cells upon detachment, which were reversed by NAC treatment or overexpression of Rac1T17N mutant, respectively (Fig. 5a, b). Conversely, H_2_O_2_ administration or Rac1 activation restored the levels of p-Src and p-FAK in IQGAP1-deficient HepG2.2.15 cells (Fig. 5a, b). We next used PP2 (a Src family kinases inhibitor) or Y15 (a specific inhibitor of FAK) to evaluate the effects of Src/FAK signalling on IQGAP1-mediated anoikis resistance. The efficiency of these inhibitors was validated by immunoblot analysis (Supplementary Fig. S6). IQGAP1 overexpression triggered the elevated anchorage-independent growth of HCC cells after detachment was alleviated significantly by PP2 or Y15 treatment (Fig. 5c, d). Consistently, PP2 or Y15 treatment effectively reversed IQGAP1-induced the reduced cleavage of caspase-3 and PARP (Fig. 5e). Similar results were obtained from transwell assay (Fig. 5f). Moreover, HepG2.2.15 cells transfected with siRNA targeting Src or FAK showed decreased cell survival in the presence of IQGAP1 (Supplementary Fig. S7a–d). Silence of Src or FAK was able to prevent enhanced migratory and invasive capacities caused by IQGAP1 in HepG2.2.15 cells (Supplementary Fig. S7e). These data imply that the activation of Src/FAK pathway is essential for IQGAP1-mediated anoikis resistance and metastasis in HCC cells.

### IQGAP1 promotes anoikis resistance and metastasis in vivo

**Fig. 6:**
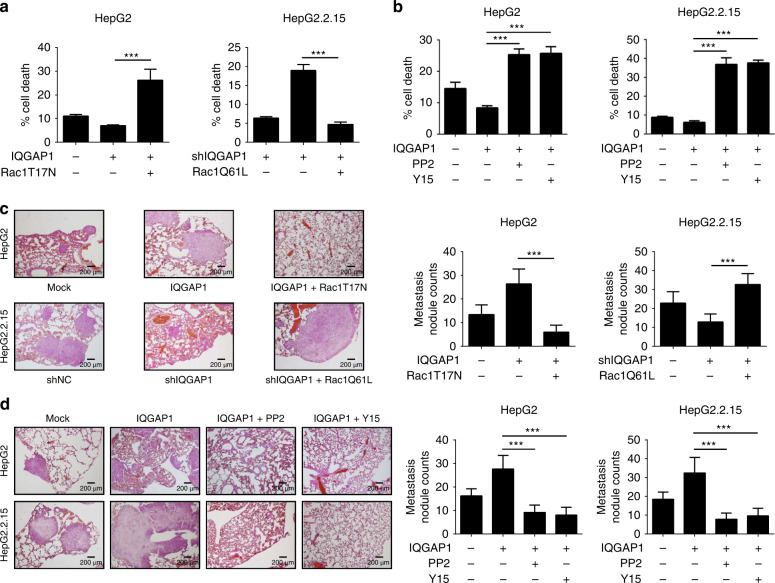
IQGAP1 enhances anoikis resistance and metastasis of HCC cells in vivo. **a** Indicated HCC cells transfected with Rac1T17N or Rac1Q61L plasmid were intraperitoneally injected into nude mice. The GFP-positive HCC cells were collected from ascites fluid and sorted by flow cytometer. The apoptotic rates of the tumour cells were counted with the trypan blue exclusion assay. **b** Effect of PP2 or Y15 on IQGAP1-mediated anoikis resistance in mouse peritoneal cavities model. **c** Indicated HCC cells were transfected as in **a**, and i.v. injected via tail veins of BALB/c nude mice. Photographs show H&E staining of dissected lung tissues 8 weeks after inoculation. **d** Effect of PP2 or Y15 on IQGAP1-mediated anoikis resistance in experimental mouse lung metastasis model. The results were expressed as mean ± SD from at least three independent experiments. ***P < 0.001.

To illustrate the effect of IQGAP1 on anoikis resistance in vivo, the GFP expressed HCC cells were intraperitoneally injected into nude mice. At the appropriate time, tumour cells were collected by flow cytometer and were subjected to trypan blue exclusion assay. As shown in Fig. 6a, IQGAP1-overexpressing HepG2 cells displayed lowered apoptosis than control cells, which were reversed by introduction of dominant-negative Rac1T17N mutant. Oppositely, IQAGP1-knockdown inhibited the anchorage-independent growth of HepG2.2.15 cells, and these affects were inverted by overexpression of constitutively active Rac1Q61L mutants (Fig. 6a). Moreover, disruption of Src or FAK activation effectively reversed the effect of IQGAP1 on anoikis resistance in vivo (Fig. 6b). To further determine if IQGAP1 can enhance the metastasis in vivo, indicated HCC cells were injected into the tail vein of nude mice. Rac1T17N mutant abrogated IQGAP1-enhanced lung metastatic potential of HepG2 cells (Fig. 6c). However, Rac1Q61L mutant restored the ability of IQGAP1-deficient HepG2.2.15 cells to establish metastases (Fig. 6c). In addition, we also confirmed that Src/FAK pathway activation was necessary for IQGAP1-mediated metastasis in vivo (Fig. 6d). These data indicate that IQGAP1 promotes anoikis resistance and metastasis via Rac1/Src/FAK axis in vivo.

## Discussion

Accumulating evidence indicates that HBV can orchestrate the activity of cytoskeleton-associated protein of host cells for virus infection, replication and dissemination.^8,33,34^ IQGAP1 is an evolutionarily conserved multifunction scaffold protein that regulates cytoskeleton remodelling, cellular motility and cell survival. Thus, IQGAP1 has been implicated in varied virus pathogenesis, including HIV, Ebola and Marburg virus.^35–37^ In the present study, we reported that IQGAP1 levels were upregulated in HBV-positive HCC tissues compared with HBV-negative HCC tissues, and higher expression of IQGAP1 was associated with poor prognosis of HBV-associated HCC patients. IQGAP1 expression was positively correlated with the expressions of HBsAg and AFP, tumour size and number, and BCLC stage. Moreover, overexpression of IQGAP1 significantly increased the apoptotic tolerance of HCC cells upon detachment, whereas IQGAP1 suppression resulted in reduced anchorage-independent growth, migratory and invasive capacities of HBV-producing HCC cells. Together, our data implicated IQGAP1 as a potential marker for HCC aggressiveness and a favourable predictor for HBV-associated HCC patients’ survival.

Oxidative stress is one of the vital factors involved in HBV-associated HCC development.^38^ A recent study demonstrated that HBV-triggered ROS accumulation resulted in Snail-mediated epigenetic silence of SOCS3 that activated IL-6/STAT3 signalling pathway to accelerate hepatocarcinogenesis.^24^ Furthermore, reduced expressions of antioxidant molecules including catalase and superoxide dismutase were associated with poor prognosis in HBV-related HCC patients.^14,39^ Here, we reported that moderate levels of ROS rescued the anchorage-independent growth, migration and invasion in IQGAP1-deficient HCC cells, suggesting that ROS were key downstream regulators of IQGAP1. Our results further support that ROS can act as essential second messengers in various signalling pathways that tune cell proliferation and survival. As excess ROS levels can cause oxidative damage and cell death, cancer cells need to appropriately modify intracellular ROS levels to survive during metastatic progression. Here, we confirmed that HBV-induced ROS enhanced the binding of IQGAP1 to Rac1, leading to stabilising the active GTP-bound form of Rac1, and ultimately activation of Rac1. Moreover, our data also showed that active Rac1 was required for IQGAP1-mediated ROS generation. In addition, it has been shown that Rac1 interacted directly with p67phox, which in turn contributed to the assembly of NADPH oxidase complexes, resulting in the production of ROS.^40,41^ Based on these observations, we speculated a positive-feedback loop developed between ROS levels and Rac1 activity in HBV-associated HCC during detachment. Hence, disruption of this feedback loop may be a potential therapeutic strategy to prevent HCC progression. Indeed, antioxidant NAC treatment significantly abolished ROS production and Rac1 activity, leading to increased apoptosis of HCC cells upon detachment.

FAK is a non-receptor protein tyrosine kinase that localises to focal adhesions and plays a critical role in many physical processes, including cell attachment, growth, metastasis and apoptosis.^42,43^ Src has been found to interact with FAK and facilitate the phosphorylation and activation of FAK.^30,44^ Here, we identified that the activation of Rac1 caused by IQGAP1 triggered the phosphorylations of Src and FAK, resulting in anoikis resistance and metastasis. Moreover, we also found the elevated ROS levels were required for activation of Src and FAK. In accordance with our findings, a recent research revealed that ROS oxidised Src on two cysteine residues (C245 and C487), thereby enhancing Src activity in prostate cancer cells.^45^ In addition, tyrosine phosphatase LMW-PTP was oxidised and inactivated during cell adhesion, leading to preventing the enzyme from dephosphorylating and inactivating FAK.^46^ Inhibition of NADPH oxidase activity decreased the association of FAK/Src complex and FAK^Y397^ phosphorylation in human melanoma cells.^47^ These observations suggest that Src/FAK signalling may act as redox sensors to integrate cell survival and metastasis. Conversely, inactivation of Src or FAK notably blocked IQGAP1-induced anchorage-independent growth and motility of HCC cells. As anoikis escape provides a selective advantage of cancer cells to distant dissemination and colonisation, our data suggest that interruption of the IQGAP1/Rac1/Src/FAK pathway might be effective for suppressing tumour growth and metastasis in chronically HBV-infected patients.

In conclusion, our current study demonstrates that high level of IQGAP1 positively correlates with aggressive HCC phenotypes and poor clinical outcome of HBV-associated HCC patients. We delineate a molecular mechanism by which HBV-induced IQGAP1 expression increased Rac1 activity and ROS accumulation that activates Src/FAK pathway, leading to enhanced anchorage-independent growth and metastasis of HCC cells. Taken together, our findings highlight the significance of IQGAP1 in HBV-mediated HCC progression and implicate IQGAP1 as a promising biomarker for the individualised management of patients with HBV-associated HCC.

## Supplementary information

Supplementary files

## Data Availability

The datasets generated and/or analysed during the current study are not publicly available but are available from the corresponding author on reasonable request.
