# Supplementary material for: IQGAP1 promotes anoikis resistance and metastasis through Rac1-dependent ROS accumulation and activation of Src/FAK signalling in hepatocellular carcinoma
Source: Br J Cancer. 2020 Jul 7;123(7):1154–63. doi: 10.1038/s41416-020-0970-z (PMC7525663; doi:10.1038/s41416-020-0970-z)
Supplement: Supplementary file 1 — Supplementary files [file 41416_2020_970_MOESM1_ESM.docx]

**
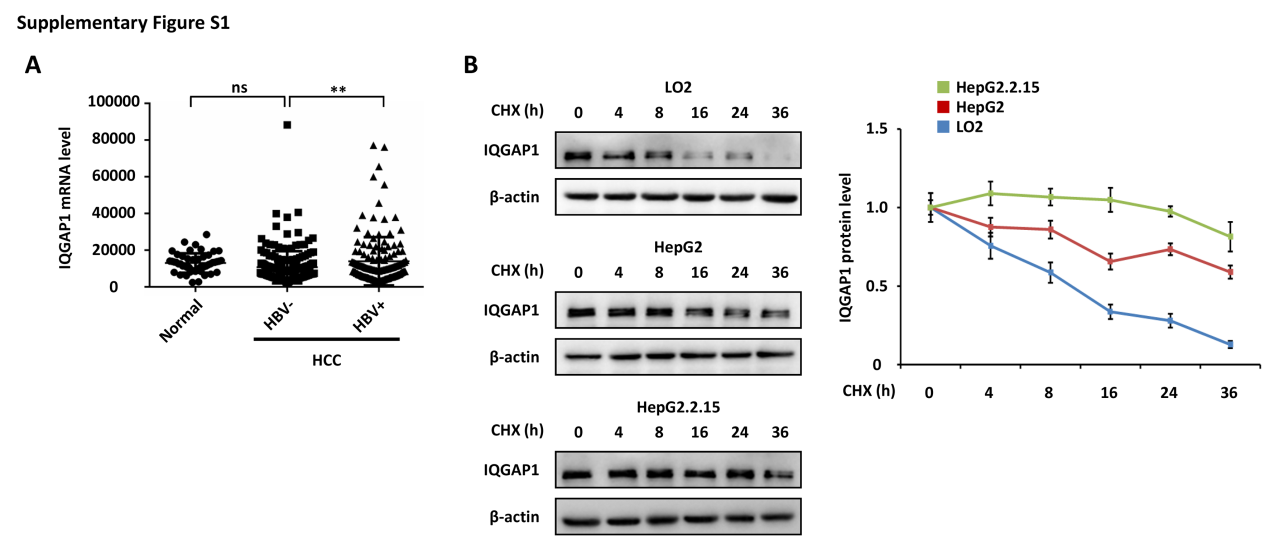
Supplementary Figure S1 HBV infection promoted IQGAP1 protein stability.** (A) The mRNA expression of IQGAP1 in TCGA liver cancer dataset. (B) Immortal hepatocyte cell line LO2, HCC cells HepG2 and HepG2.2.15 were treated with Cycloheximide for indicated time, and the degradation rate of IQGAP1 protein was examined by western blot. Each experiment was performed at least in triplicate, producing consistent results. ns, no significant, and ***P* < 0.01

**
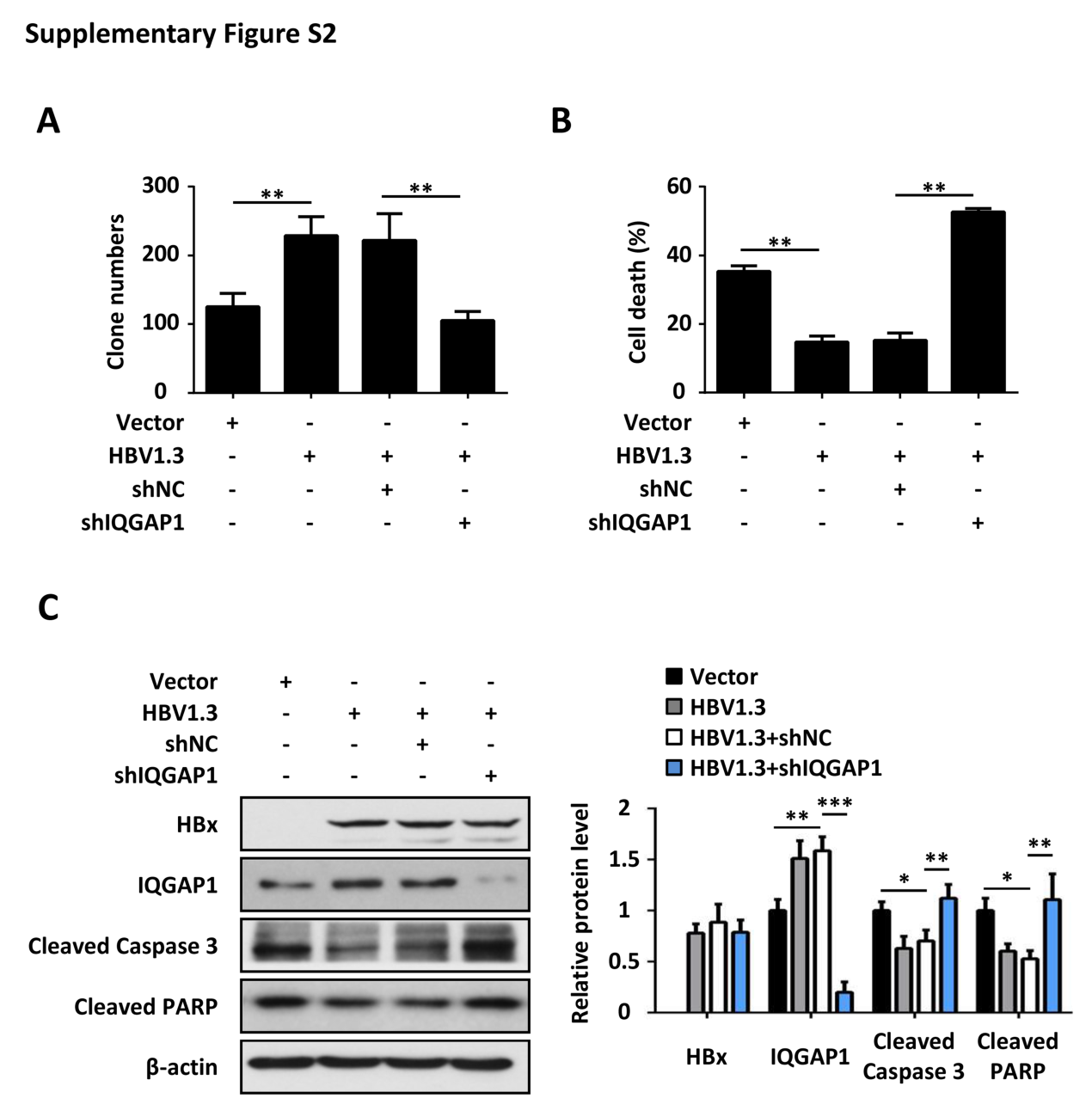
**

**Supplementary Figure S2 IQGAP1 is essential for HBV-mediated anoikis resistance.** (A) Huh7 cells were transfected with HBV1.3 plasmid in the presence or absence of shIQGAP1 lentiviral infections. The anchorage-independent growths of indicated Huh7 cells were determined by soft agar colony formation assays. (B) The cell death of indicated Huh7 cells under suspension condition was determined by trypan blue exclusion assay. (C) The indicated Huh7 cells were cultured in suspension condition and then subjected to immunoblot analysis using the indicated antibodies. Data represent the mean ± SD. Each experiment was performed at least in triplicate, producing consistent results. ***P* < 0.01


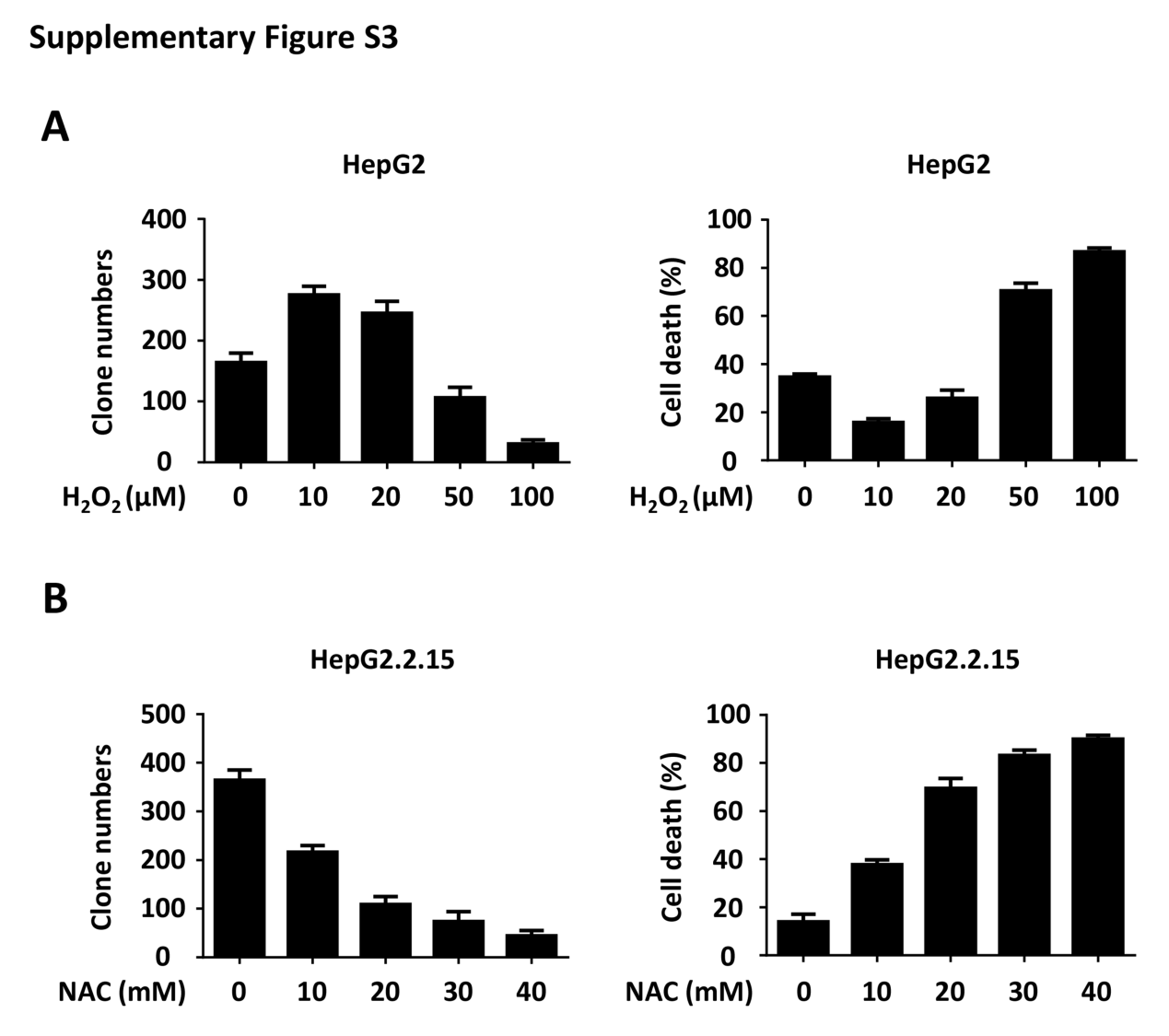


**Supplementary Figure S3 ROS levels regulate anoikis of HCC cells.** (A) HepG2 cells were treated with various concentration of H_2_O_2_, and then subjected to soft agar colony formation and trypan blue assays. (B) HepG2.2.15 cells were treated with various concentration of NAC, and then subjected to soft agar colony formation and trypan blue assays. Data represent the mean ± SD. Each experiment was performed at least in triplicate, producing consistent results.


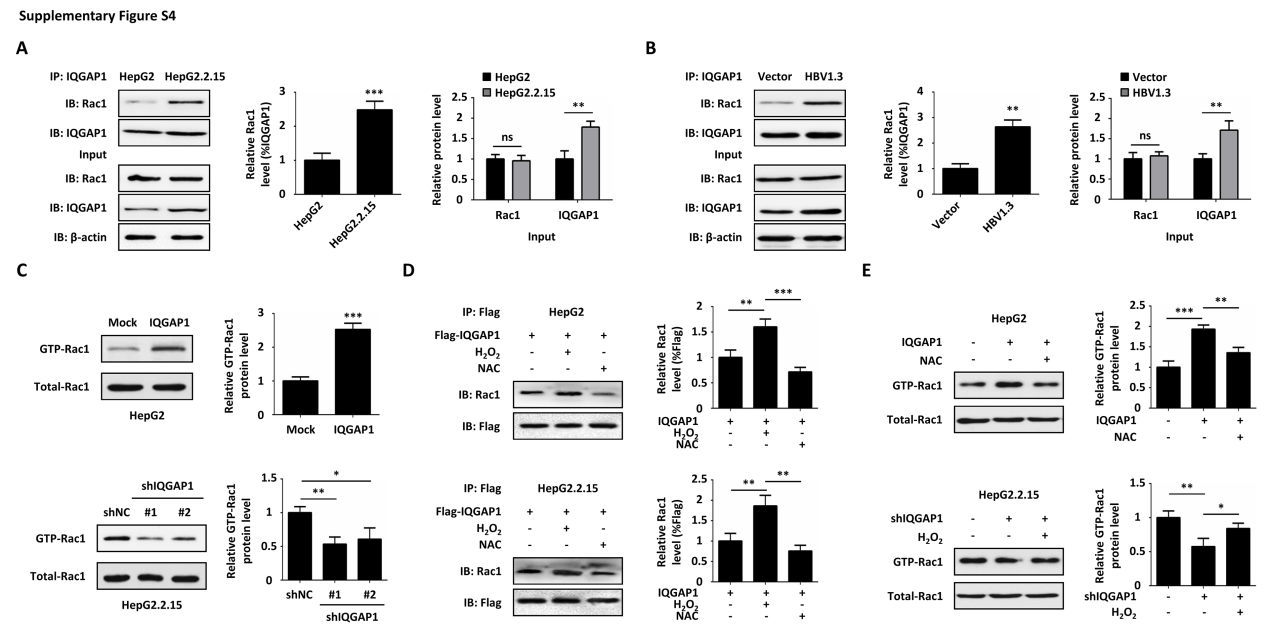


**Supplementary Figure S4** **HBV-induced ROS enhances association of IQGAP1 with Rac1 that activates Rac1.** (A) The endogenous interaction of IQGAP1 and Rac1 in HepG2 and HepG2.2.15 cells was detected by immunoprecipitation with anti-IQGAP1 antibody. (B) The impact of HBV on the interaction of IQGAP1 and Rac1 in Huh7 cells. (C) Protein lysates prepared from indicated HepG2 and HepG2.2.15 cells. GTP-bound Rac1 was precipitated from lysates by incubation with GST-PAK1-PBD and resolved by SDS-PAGE. Representative Western blots are shown. (D) HepG2 and HepG2.2.15 cells transfected with Flag-IQGAP1 plasmid were treated with 20 mM NAC or 10 μM H_2_O_2_, respectively, and the association of IQGAP1 and Rac1 was detected by immunoprecipitation with indicated antibody. (E) Indicated HepG2 and HepG2.2.15 cells cultured in suspension were treated with 20 mM NAC or 10 μM H_2_O_2_, respectively, and then Rac1 activity assay was performed. Each experiment was performed at least in triplicate, producing consistent results.


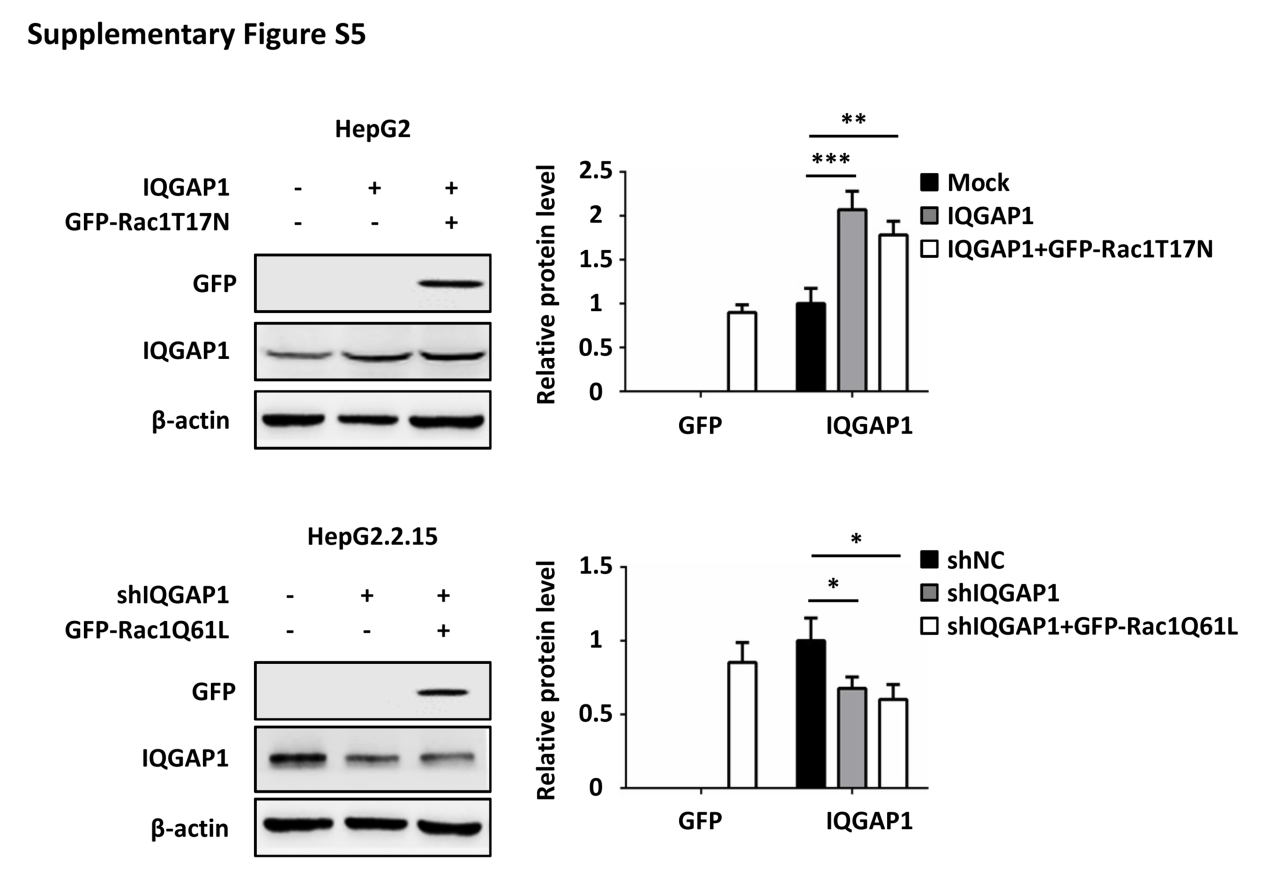


**Supplementary Figure S5 The validation of Rac1 plasmids in HCC cells.** IQGAP1 overexpressing HepG2 cells were transfected with Rac1T17N plasmid and IQGAP1-deficient HepG2.2.15 cells were transfected with Rac1Q61L plasmid, respectively. Immunoblot analysis was performed using the indicated antibodies. Each experiment was performed at least in triplicate, producing consistent results.


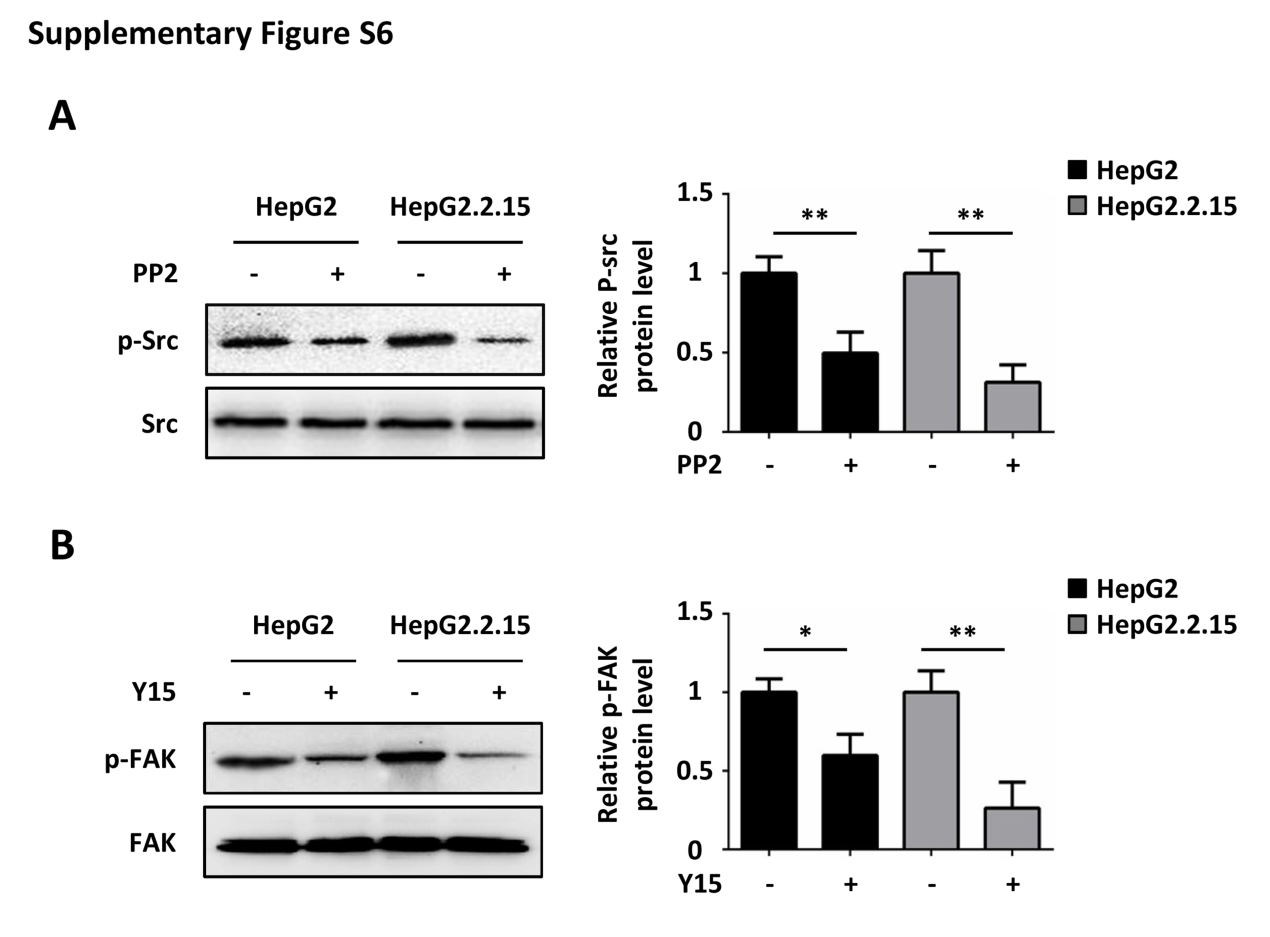


**Supplementary Figure S6 The effect of PP2 and Y15 on the phosphorylation of Src and FAK, respectively.** HepG2 and HepG2.2.15 cells were treated with PP2 (A) or Y15 (B), and subjected to immunoblot analysis using the indicated antibodies. Each experiment was performed at least in triplicate, producing consistent results.


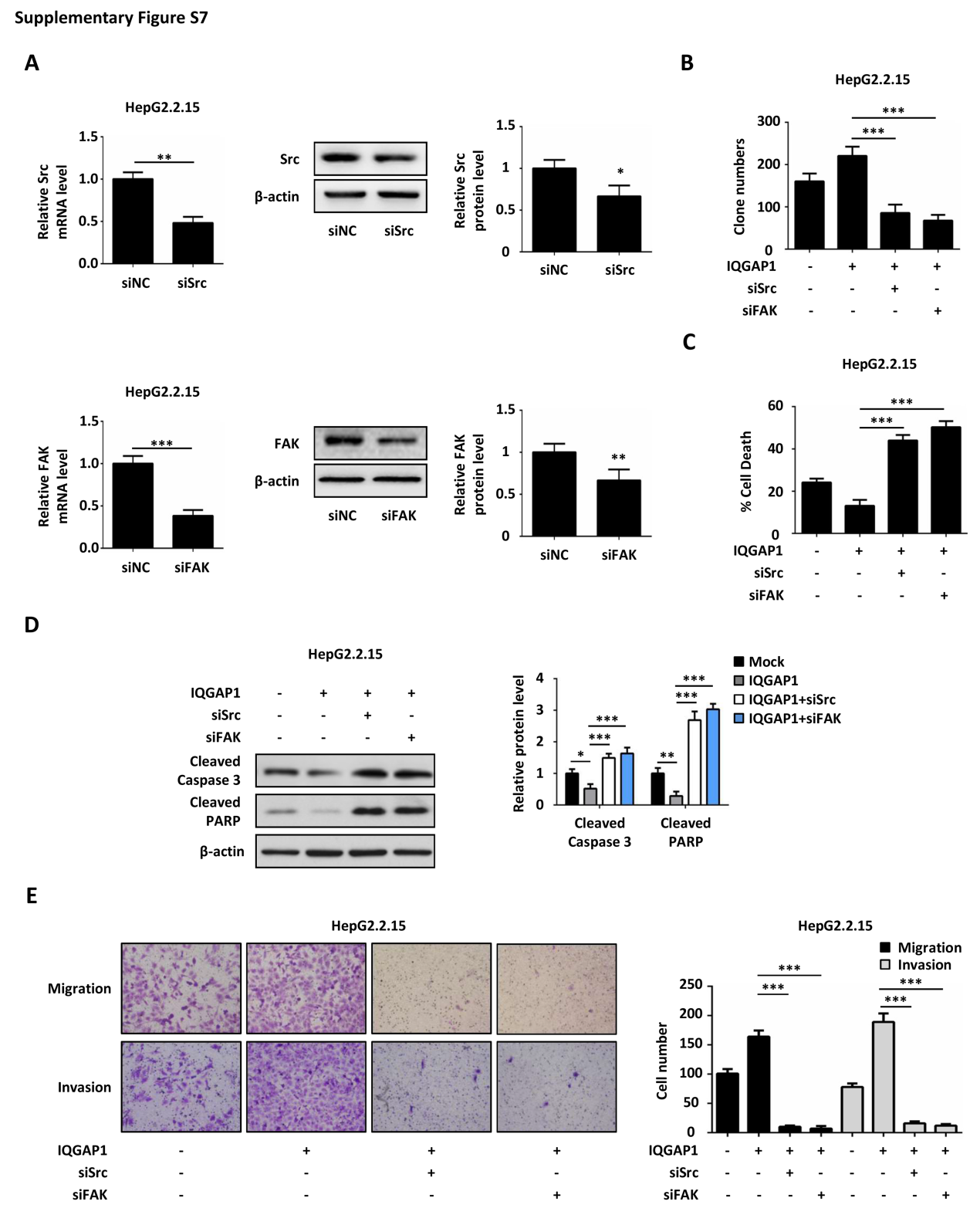


**Supplementary Figure S7 Src/FAK signaling is indispensable for IQGAP1-mediated anoikis resistance and metastasis in HepG2.2.15 cells.** (A) The knockdown of Src or FAK in HepG2.2.15 cells was confirmed by qRT-PCR and western blot. (B) Indicated HepG2.2.15 were transfected with siRNA targeting Src or FAK, and then subjected to soft agar colony formation assays. (C) Indicated HepG2.2.15 cells were transfected with siRNA targeting Src or FAK, cultured in suspension condition, and then subjected to trypan blue assay. (D) Indicated HepG2.2.15 cells were transfected, cultured as in (C), and subjected to immunoblot analysis using the indicated antibodies. (E) Indicated HepG2.2.15 cells were transfected with siRNA targeting Src or FAK, and then subjected to transwell migration and invasion assays. Cells were counted under a light microscope at five random fields. Data represent the mean ± SD. Each experiment was performed at least in triplicate, producing consistent results. ***P* < 0.01 and ****P* < 0.001.
